# Neuromyelitis optica: a pilot study of clinical presentation and status of serological biomarker AQP4 among patients admitted to a tertiary centre in NCNS, Sudan

**DOI:** 10.1186/s12868-020-0557-x

**Published:** 2020-02-28

**Authors:** Etedal Ahmed AbuElbasher Ibrahim, Fatima Gammer, Alsadig Gassoum

**Affiliations:** 1 Alneelain University, Faculty of Medicine, Khartoum, Sudan; 2 The National Center for Neurological Sciences, Khartoum, Sudan; 3 ALMadain College, Khartoum, Sudan

**Keywords:** AQP4-IgG, Neuromyelitis optica, Serological biomarkers, Sudan

## Abstract

**Background:**

Neuromyelitis optica (NMO) is a demyelinating disease primarily affecting the optic nerves and spinal cord. It is distinguished from other demyelinating conditions by the presence of AQP4-IgG and serum aquaporin 4 (AQP4), found mainly in the blood–brain barrier. This descriptive study was conducted from January 2015 to June 2018 at the National Center for Neurological Sciences (NCNS) in Khartoum, Sudan. All participants were Sudanese patients diagnosed with NMO. In our study the selection of cases was based on Dean Wingerchuk diagnostic criteria (2006), which states that the diagnosis of NMO should meet two absolute criteria and two supportive criteria. The absolute criteria are myelitis and optic neuritis, whereas supportive criteria include radiological findings obtained from brain and spinal cord MRI. Furthermore, AQP4-IgG levels were measured from cerebrospinal fluid (CSF) and serum using immunofluorescence. Data were collected by a pre-designed questionnaire and analyzed using SPSS version 17. A p value < 0.05 was considered statistically significant.

**Results:**

A total of 31 patients were enrolled in this study [6 male (19.4%) and 25 female (80.6%)]. The mean age was 38 ± 12.8 years. Motor and visual difficulties were the initial symptoms and occurred in 21 (67.7%) and 10 (32.3%) patients, respectively. Fundoscopy confirmed optic atrophy in 22 (71.0%) patients. The course of the disease revealed one relapse in 21 patients (67.7%). Seropositive AQP4-IgG were seen in 23 patients (79.31%). There was a significant correlation between AQP4 and response to treatment (p ≤ 0.038). The correlation between serum AQP4-IgG, showed that, complete improvement was detected in 2 patients (6.9%) one of them was positive and the other was negative, 20 (69.0%) patients presented with some disability, among them 18 (62.1%) were positive and 2 (6.9%) were negative, while 7 patients showed no improvement (24.1%) 4 out of them were positive (13.8%) and 3 were negative (10.3%).

**Conclusion:**

At the initial presentation of NMO, longitudinal myelitis was observed more frequently than optic neuritis. More than two third of the patients showed strong seropositivity for serum AQP4. Most seropositive patients showed a good response to treatment but with residual disabilities.

## Background

Neuromyelitis optica (NMO) is a demyelinating disease primarily affecting the optic nerves and spinal cord [1]. It is characterized by the presence of optic neuritis and/or myelitis. It can be monophasic but 73% to 90% of cases have a relapsing course [2].

The disease is distinguished from other demyelinating conditions by the presence of AQP4-IgG which is expressed by the gene AQP4, and is mainly found in the blood–brain barrier of the central nervous system [3]. The gene belongs to the AQP4 family whose members conduct water through cell membranes [3]. Their main function is to transport water and maintain homeostasis within the nervous system. AQP4 is a predominant target in neuromyelitis optica. Detection of this protein in the serum of the patient reflects the disease activity. The diagnosis of the disease can be made by the associated clinical characteristics, MRI findings of a longitudinal lesion involving more than 3 consecutive vertebrae, and the presence of AQP4-IgG in the CSF as well as positive serum AQP4 [4–10].

### Objective

To study the clinical presentation and serological biomarker status and disease outcome and its correlation with AQP4 among Sudanese patients at the National Center of Neurological Sciences (NCNS) in Khartoum, Sudan, from January 2015 to June 2018.

## Methods

This was a descriptive study. The study included 31 NMO patients with diagnosis based on the following: clinical presentation with optic neuritis and myelitis confirmed by MRI of the brain with FLAIR and MRI of cervical and dorsalspine, as well as positivity of serum Aquaporin 4 and CSF for AQP4-IgG (performed using ELISA RSR Ltd, Cardiff, UK). Other investigations were done to exclude mimickers such as: serum and CSF oligoclonal bands to rule out multiple sclerosis (MS), complete blood count, erythrocyte sedimentation rate (ESR), C-reactive protein, anti-nuclear antibody (ANA), anti- double-stranded DNA antibodies (Anti-dsDNA), and ACLP antibodies to exclude vasculitis. The blood and CSF samples were analyzed by immunofluorescence.

In our study the selection of cases was based on Dean Wingerchuk diagnostic criteria (2006), which require that patients meet both the two absolute criteria and any two of the supportive criteria [11].

### Absolute criteria

1. Optic neuritis.
2. Acute myelitis.

### Supportive criteria

1. MRI of the brain not meeting the criteria for MS at disease onset.
2. MRI of the spinal cord with contiguous T2-weighted signal abnormality extending over three or more vertebral segments, indicating a relatively large lesion in the spinal cord.
3. AQP4-IgG seropositive status (The AQP4-IgG test checks the existence of antibodies against the Aquaporin 4 antigen).

Data was collected by a comprehensive structured questionnaire. It covered demographics and medical data in addition to descriptive statistics in terms of frequency, and tables with percentages for the qualitative data. Data was collected, reviewed and analyzed using SPSS version 17. Furthermore; relations were analyzed using cross-tabulation and Chi square test. A *p* value of < 0.05 was considered statistically significant.

The study was ethically approved by the National Center for Neurological Sciences ethical committee, Khartoum, Sudan. Written consent was taken from all the patients who participated in the study.

## Results

### Demographic characteristics

A total of 31 Sudanese patients with NMO were included in this study. Six (19.4%) of the patients were males and 25 (80.6%) were females. Seventeen (54.8%) patients were more than 40 years and the mean age was 38.74 ± 12.85 years. In this study the distribution of NMO patients indicated 18 (58.1) patients from the north of Sudan, the majority of the tribes which reside in this area were belonging to Afro-asiatic group, while 8 (25.8) from eastern Sudan, which were belonging also to Afro-asiatic group, and 5 patients (16.1%) from the western Sudan, the most common tribes in this state are from Nilo-Saharan group. From our data registry we found that, the total number of NMO patients during the last year was 38, and accordingly the incidence will be 0.81.

Motor symptoms were reported first in 21 patients (67.7%) and visual symptoms in 10 patients (32.3%). Among the 10 patients with visual symptoms, 8 of them (80.0%) had complete visual loss and more frequent in bilateral optic neuritis, the frequency of bilateral optic neuritis was 41.9% and unilateral optic neuritis represented 19.4%, 1 (10%) had impaired visual acuity, and 1 (10%) had impaired acuity as well as color vision. Among the 21 patients with motor symptoms, 19 (90.5%) had lower limb weakness, 1 (4.76%) had upper limb weakness, and 1 (4.76%) patient had both. Seventeen (54.8%) patients had sensory symptoms while 12 (38.7%) had sphincteric disturbances. Table 1 describes these findings.

**Table 1:** Demographic characteristics of the study participants

Variables	Findings	No	Percentage (%)
Gender	Male	6	19.4
	Female	25	80
Age in years	16–20	4	12.9
	21–25	3	9.7
	26–30	3	9.7
	31–35	3	9.7
	36–40	1	3.2
	> 40	17	54.8
Symptoms	Visual	10	32.3
	Motor	21	67.7
Visual status	Impairment of visual acuity	1	10
	Complete visual loss	8	80
	Impairment of visual acuity & color vision	1	10
Disease duration since the onset	Days	5	16.1
	Weeks	6	19.4
	Months	10	32.3
	Years	10	32.3
Visual acuity	Blindness	13	41.9
	Normal	12	38.7
	Impaired	6	19.4
Fundus examination	Optic atrophy	22	71.0
	Normal	9	29.0
Motor system findings	Paraparises	6	19.4
	Paraplegia	16	51.6
	Quadriparesis	4	12.9
	Quadriplegia	5	16.1
	Complete improvement	6	19.4
Clinical course	Single relapse	21	68
	Relapsing	10	32
MRI brain	Normal	23	74
	Abnormal	8	26
MRI spinal cord	Normal	2	6
	Abnormal	29	94
CSF NMO-IgG	+Ve	25	79
	−Ve	6	21
Serum aquaporin-4 antibody	+Ve	25	79
	−Ve	6	21
Treatment	IV methylprednisolone followed by oral	5	16
	IV methylprednisolone followed by oral+ azathioprine	26	84
Outcomes	Complete improvement	2	6
	Improvement with disability	22	71
	No improvement	7	23

The interval between visual and motor symptoms varied between days, weeks, months, and years in 5 (16.1%), 6 (19.4%), 10 (32.3%), and 10 (32.3%) patients, respectively. Twenty-one patients (67.7%) had one attack whereas 10 (32%) had a history of many attacks (Table 1).

**Fig. 1:**
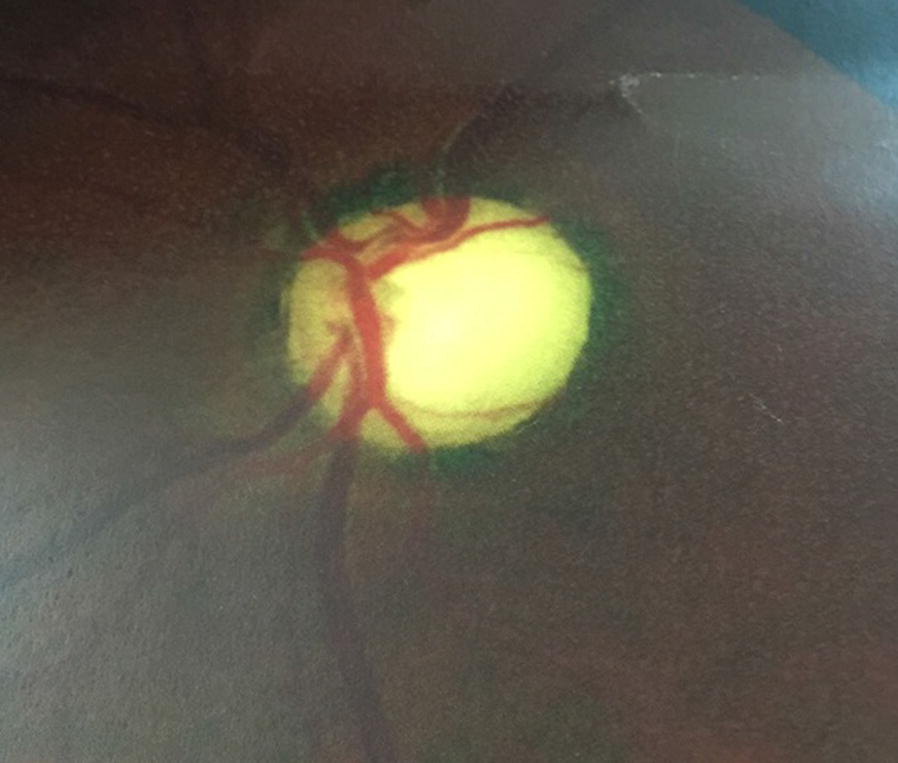
Optic atrophy in a patient with NMO presented with reduce vision both eyes and lower limbs weakness, over 2 month duration

Interestingly, one patient (3.2%) had a family history of NMO. All patients (100%) had normal higher-order functions. Among the visual symptoms, 13 (41.9%) patients had blindness and 6 (19.4%) patients had impaired visual acuity while 12 (38.7%) had normal visual acuity. Seventeen patients (54.8%) had normal color vision in contrast to 14 (45.2%) patients who had abnormal color vision. In addition, 17 (54.8%) of the patients had central scotoma. Fundoscopy showed that 22 (71.0%) of the patients had optic atrophy (Fig. 1) while the rest had normal optic discs. Other cranial nerves were normal in 28 patients (90.3%) but were found to be abnormal in 3 (9.7%) (Table 1).

Motor system impairments ranged from paraparesis which was observed in 6 (19.4%) patients to paraplegia in 16 patients (51.6%), quadriparesis in 4 (12.9%) patients. and quadriplegia in 5 patients (16.1%). Among patients with a relapsing course, 5 patients (50.0%) had 2 attacks, 4 patients (40%) had three attacks, and 1 patient (10%) had more than three attacks. We did not find any previous registered data regarding the prevalence of NMO in Sudan. Simultaneously, we studied 65 patients with demyelinating disease (multiple sclerosis) in Sudan (2018) [12]. According to disability score, 14 patients EDSS score 8, 9 patients score 9, 5 patients score 7.5, 1 patient score 5, 1 patient score 4 and 1 patient score 3.

**Fig. 2:**
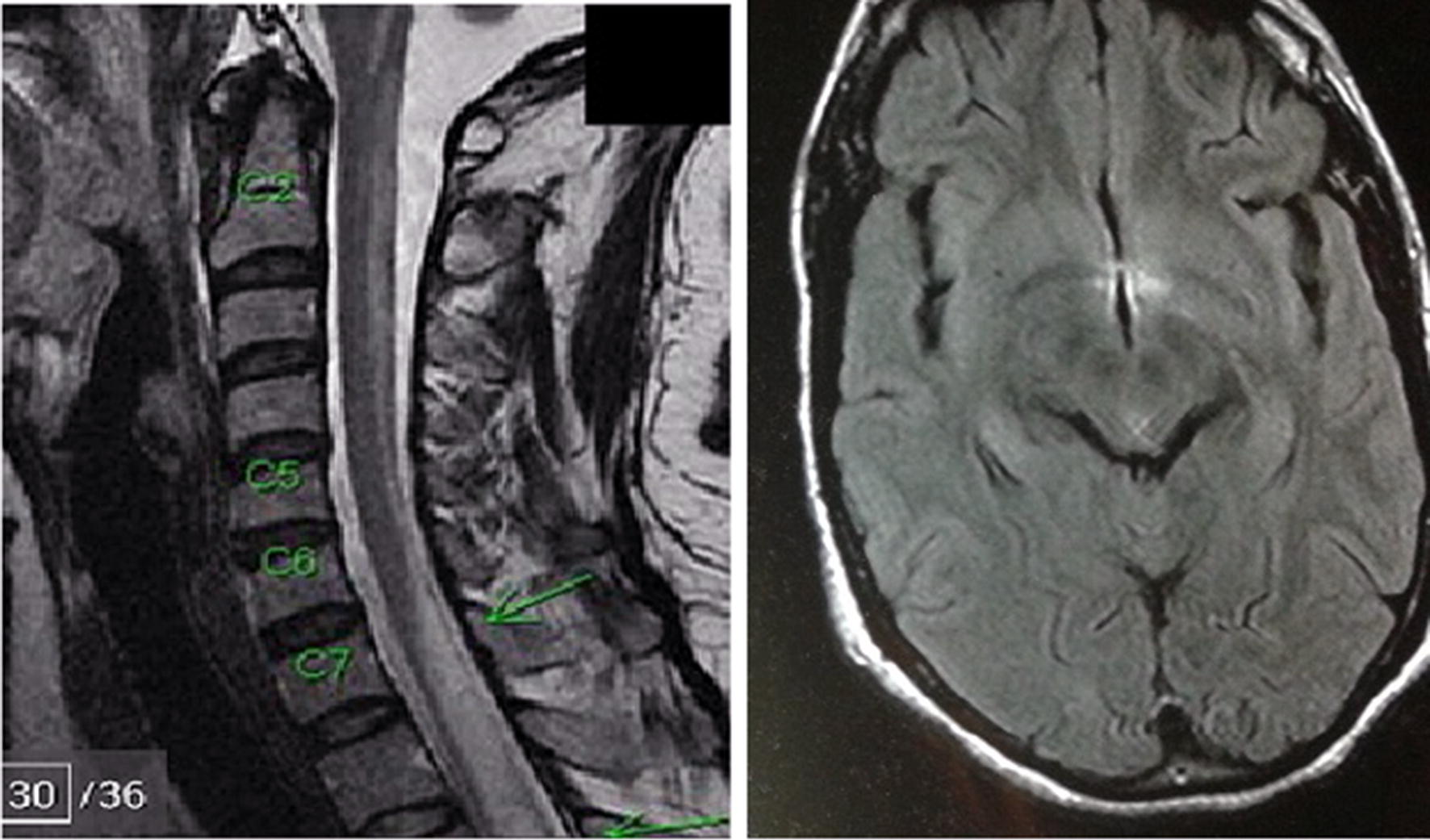
MRI of the brain showing hyper-intensity in the aquaporin4 area, and MRI of the cervical spine showing longitudinal hyper-intensities in more than 3 consecutive vertebrae in a patient with NMO presented with reduce vision both eyes and lower limbs weakness, over 2 month duration

MRI of the brain was normal in 23 patients (74.2%); however it was abnormal in 8 patients (25.8%) as exemplified in Fig. 2. Similarly, MRI of the cervical spine was normal in 2 patients (6.5%) but was abnormal in 29 patients (93.5%); it showed longitudinal hyper-intense lesion involving more than 3 consecutive vertebrae (Table 1).

1. In this study when CSF analysis was performed in 31 patients it was positive for AQP4-IgG in 23 patients (79.31%) and negative in 6 patients (20.69%). The results of serum Aquaporin4 were positive in 23 patients (79.31%) and negative in 6 patients (20.29%), those 6 patients in the study with negative CSF AQP4-IgG were also negative for serum AQP4-IgG. Oligoclonal bands were negative in 29 patients (93.5%). The correlation between serum AQP4-IgG, showed that, complete improvement was detected in 2 patients (6.9%) one of them was positive and the other was negative 20 (69.0%) patients presented with some disability, among them 18 (62.1%) were positive and 2 (6.9%) were negative, while 7 patients showed no improvement (24.1%) 4 out of them were positive (13.8%) and 3 were negative (10.3%).

A majority of the studied population (26, 83.9%) received treatment in the form of intravenous methylprednisolone 1 gm/day for 5 days followed by oral prednisolone 60 mg/day tapered down according to the response combined with azathioprine 150 mg/day while 5 patients (16.1%) did not receive azathioprine.

Among the patients, 22 (71.0%) showed improvement with some remaining disability, whereas 2 patients (5.6%) showed complete recovery and 7 (22.6%) demonstrated no improvement (Table 1). The results of correlation between the outcome and serum AQP4are displayed in Table 2, and those of the correlation between treatment and serum AQP4 are displayed in Table 3.

**Table 2:** Correlation between outcome and serum aquaporin-4 antibody among the study population

Outcome		Serum aquaporin-4 antibody		Total
		+ve	−ve	
Complete improvement	Count	1	1	2
	% of total	3.4%	3.4%	6.9%
Improvement with some disability	Count	18	2	20
	% of total	62.1%	6.9%	69.0%
No improvement	Count	4	3	7
	% of total	13.8%	10.3%	24.1%
Total	Count	23	6	29
	% of total	79.3%	20.7%	100.0%

P-value = 0.104

**Table 3:** Correlation between treatment and serum aquaporin-4 antibody among the study population

Treatment		Serum aquaporin-4 antibody		Total
		+ve	−ve	
IV methylprednisolone followed by oral prednisolone	Count	1	2	3
	% of total	3.4%	6.9%	10.3%
IV methylprednisolone followed by oral prednisolone + azathioprine	Count	22	4	26
	% of total	75.9%	13.8%	89.7%
Total	Count	23	6	29
	% Of Total	79.3%	20.7%	100.0%

P-value = 0.038

Figure 1 shows optic atrophy in a 37-year-old Sudanese female with NMO.

Figure 2 shows MRI of the brain with hyper-intensity in the area of AQP4, and MRI of the cervical spine with longitudinal hyper-intensity involving more than 3 consecutive vertebrae.

## Discussion

Our study is the first comprehensive attempt to estimate the frequency of NMO and AQP4-IgG seropositivity in Sudanese patients. NMO seemed to be a rare disease in Sudan but our study suggests that it is probably common. In this study, females were more often affected (80.6% of the study population). This finding is similar to those of previous studies from Caucasians, French West Indies, Austria and Cuba that estimated female predominance in 66–88% of the patients [13–16].

Our study revealed that 17 patients (54.8%) were above the age of 40 years. A study from Cuba described a mean age of 35 years [17] whereas other studies reported different mean age of patients [13, 15, 18].

The distribution of NMO patients in this study indicated, 18 (58.1) patients came from the north of Sudan, and the majority of the tribes which reside in this area were belonging to Afro-asiatic group, while 8 (25.8) from eastern Sudan, which were belonging also to Afro-asiatic group, and 5 patients (16.1%) from the western Sudan, the most common tribes in this state are from Nilo-Saharan group.

In our study, motor symptoms secondary to myelitis were observed more often than visual symptoms due to optic neuritis (67.7% vs. 32.2% respectively). Among the 10 patients with visual symptoms, 8 of them (80.0%) had complete visual loss and more frequent in bilateral optic neuritis, the frequency of bilateral optic neuritis was 41.9% and unilateral optic neuritis represented 19.4%, 1 (10%) had impaired visual acuity, and 1 (10%) had impaired acuity.

This is similar to the findings from previous studies in which the presenting symptoms were usually either secondary to optic neuritis in 35% of the patients or due to transverse myelitis in 70% [19–21].

Complete visual lost during the attack is very high compared to other cohort studies, and this could be due to the late of the patient’s presentation.

The clinical course of the disease following the diagnosis was the predominant presentation of single relapse and multiple relapses in one third of the patients with the median course of the disease of 21 months.

This is comparable to previous data that reported a relapse rate of 55% in the first year [22]. In most of the seropositive patients, relapse occur early with about 55% of patients having a relapse in the first year and 90% within the first 5 years [23]. However, among the seropositive patients that we studied 68% reported a single relapse course during the 3 year follow-up. These patients were on azathioprine treatment since the onset of the disease. This might be the cause of reduction of relapses during the study duration, however further relapses may occur in future. Another explanation could be that relapses in those seropositive patients (68%) might have occurred during the disease follow-up duration as a mild relapse in addition to the persistent disability of the patients, which could have passed unnoticed by the patients.

The findings from this study demonstrate that AQP4-IgG seropositivity was high with a frequency of 79.3%, samples were analyzed using ELISA, but the cell-based assays are superior to ELISAs. These findings are in agreement with those of international studies [19–21] except a study conducted in Saudi Arabia [21]. In recent years, serum reactivity that target AQP4-IgG is detectable in 60% to 90% of patients with NMO. Some studies have indicated that CSF AQP4IgG is present in most patients with positive serum AQP4IgG. AQP4IgG is detectable mostly as a result of passive diffusion from the serum [24]. This could explain the equality of the percentage of CSF and serum AQP4-IgG.

With respect to the treatment and outcome, 26 of the patients (83.9%) received treatment in the form of intravenous (IV) methylprednisolone 1 gm/day for 5 days followed by oral prednisolone 60 mg/day tapered down according to the response, and azathioprine 150 mg/day; and a majority 22 (71.0%) of the patients improved with some disability. These results are in accordance to a previous study which stated that 69% of patients treated with oral prednisolone and azathioprine showed a marked reduction in the relapse rate [25]. That plasma exchange therapy was not available for the majority of patients at that time but now it’s available, plasma exchange is very effective in acute relapse of neuromyelitis optica [26]. In Sudan we can deal with the approval of the first expensive NMOSD therapy (Eculizumab) in the future, although most of our patients are of low socioeconomic status.

In our study, there was a significant correlation between the response to treatment and serum Aquaporin4.

## Conclusion

Females were more affected than males and patients older than 40 years were over- represented. The most frequent initial presentation was motor impairment rather than optic neuritis. Neuroimaging demonstrated abnormal MRI of cervical spine in the form of longitudinal hyper-intense lesion involving more than 3 consecutive vertebrae, whilst most patients had a normal MRI of the brain. This study showed a higher seropositivity for AQP4 compared to other countries. Most of seropositive patient’s showed a good response to treatment but with residual disabilities.

## Data Availability

The datasets used and/or analyzed during the current study are available from the corresponding author upon request.

## Abbreviations

APQ4-IgG: Aquaporin 4 immunoglobulin
CSF: Cerebrospinal fluid
NCNS: National Center for Neurological Sciences
NMOSD: Neuromyelitis optica spectrum diseases
MRI: Magnetic resonance imaging
